# Author Correction: Hippocampal oxytocin receptors are necessary for discrimination of social stimuli

**DOI:** 10.1038/s41467-018-02965-y

**Published:** 2018-02-02

**Authors:** Tara Raam, Kathleen M. McAvoy, Antoine Besnard, Alexa H. Veenema, Amar Sahay

**Affiliations:** 1 Center for Regenerative Medicine, Massachusetts General Hospital, Boston, MA 02114 USA; 2 Harvard Stem Cell Institute, Cambridge, MA 02138 USA; 3 Department of Psychiatry, Massachusetts General Hospital, Harvard Medical School, Boston, MA 02114 USA; 4 Program in Neuroscience, Harvard Medical School, Boston, MA 02115 USA; 5 Department of Psychology, Michigan State University, East Lansing, MI 48824 USA; 6 Broad Institute of Harvard and MIT, Cambridge, MA 02142 USA

Correction to: *Nature Communications* 10.1038/s41467-017-02173-0, Article published online 08 December 2017

The original version of this Article contained an error in the spelling of the author Alexa H. Veenema, which was incorrectly given as Alexa Veenema. This has now been corrected in both the PDF and HTML versions of the Article.

