# Supplementary material for: Hippocampal oxytocin receptors are necessary for discrimination of social stimuli
Source: Nat Commun. 2017 Dec 8;8:2001. doi: 10.1038/s41467-017-02173-0 (PMC5722862; doi:10.1038/s41467-017-02173-0)
Supplement: Supplementary file 1 — Supplementary Information [file 41467_2017_2173_MOESM1_ESM.pdf]

Supplementary Information

Supplementary Figure 1. Characterization of *Oxtr* distribution in posterior hippocampus. (**a**) Representative low-magnification (left) and high-magnification (right) images of *Oxtr* and *Gad1* mRNA expression in posterior DG by FISH (n=3 males, 3 females). Asterisk (*) denotes *Oxtr*$^+$/*GAD1*$^+$ interneuron. Arrowhead (<) denotes *Oxtr*$^+$/*GAD1*$^-$ mossy cell. Scale bar denotes 100 µm. Inset scale bar denotes 50 µm. (**b**) Quantification of *Gad1* and *Oxtr* colocalization for males and females, expressed as a percentage of *Gad1*$^+$ cells over total *Oxtr*$^+$ cells. (**c**) Representative low-magnification (left) and high-magnification (right) images of *Oxtr*, *PV*, and *SST* mRNA expression in posterior DG by FISH (n=3 males, 3 females). Arrowhead (<) denotes *Oxtr*$^+$/PV$^+$ interneuron. Asterisk (*) denotes *Oxtr*$^+$/*SST*$^+$ interneuron. (**d**) Quantification of *Oxtr* colocalization with *PV* and *SST* for males and females, expressed as a percentage of total *Oxtr* cells. (**e**) Representative low-magnification (left) and high-magnification (right) images of *Oxtr* and *Gad1* mRNA expression in pCA2/3 by FISH (n=3 males, 3 females). Asterisk (*) denotes *Oxtr*$^+$/*GAD1*$^+$ interneuron. Arrowhead (<) denotes *Oxtr*$^+$/*GAD1*$^-$ pyramidal neuron. (**f**) Quantification of *Gad1* and *Oxtr* colocalization for males and females, expressed as a percentage of *Gad1*$^+$ cells over total *Oxtr*$^+$ cells. (**g**) Representative low-magnification (left) and high-magnification (right) images of *Oxtr*, *PV*, and *SST* mRNA expression in pCA2/3 by FISH (n=3 males, 3 females). Arrohead (>) denotes *Oxtr*$^+$/PV$^+$ interneuron. Asterisk (*) denotes *Oxtr*$^+$/SST$^+$ interneuron. (**h**) Quantification of *Oxtr* colocalization with *PV* and *SST* in pCA2/3, expressed as a percentage of total *PV* cells (top) or *SST* cells (bottom). (**i**) Representative low-magnification (left) and high-magnification (right) images of *Oxtr* and *Gad* mRNA expression in pCA1d by FISH (n=3 males, 3 females). (**j**) Quantification of mean *Oxtr* intensity for CA regions expressed in arbitrary units, normalized for background .(**k**) Representative low-magnification (left) and high-magnification (right) images of *Oxtr* and *Gad1* mRNA expression in pCA1v by FISH (n=3 males, 3 females). Asterisk (*) denotes *Oxtr*$^+$/*GAD1*$^+$ interneuron. Arrowhead (>) denotes *Oxtr*$^+$/*GAD1*$^-$ pyramidal neuron. (**l**) Quantification of *Gad1* and *Oxtr* colocalization for males and females, expressed as a percentage of *Gad1*$^+$ cells over total *Oxtr*$^+$ cells. (**m**) Representative low-magnification (left) and high-magnification (right) images of *Oxtr*, *PV*, and *SST* mRNA expression in pCA1v by FISH (n=3 males, 3 females). Arrowhead (>) denotes *Oxtr*$^+$/PV$^+$ interneuron. Asterisk (*) denotes PV+/SST+ interneuron. (**n**) Quantification of *Oxtr* colocalization with *PV* and *SST* in pCA2/3, expressed as a percentage of total *PV* cells (top) or *SST* cells (bottom).

Supplementary Figure 1

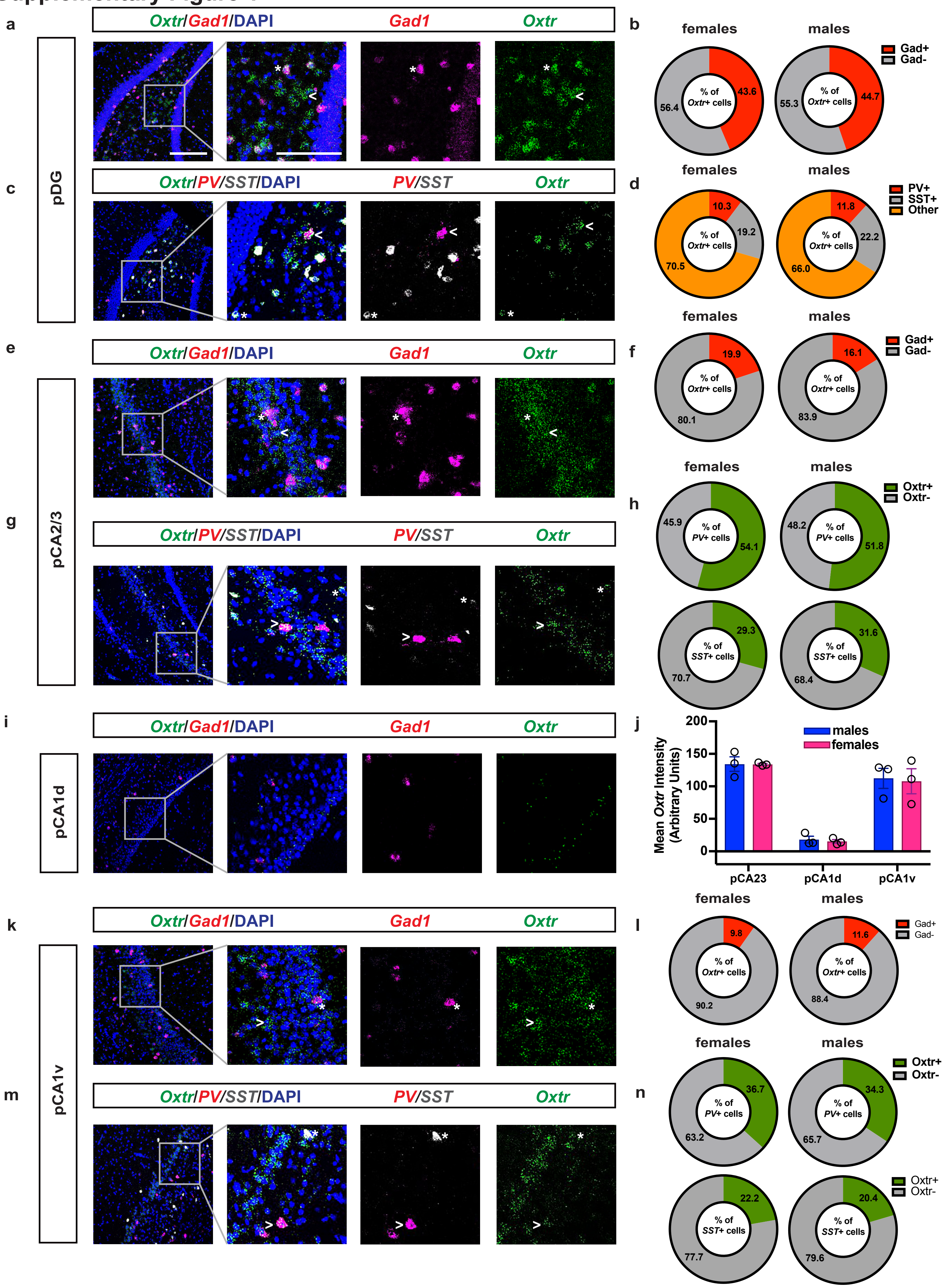

Supplementary Figure 2. AAV$_9$-hSyn-Cre-GFP injected into aDG is not retrogradely or anterogradely trafficked.

(**a**) Low magnification (top) and high magnification (bottom) images of anterior hippocampus at the site of injection in aDG. Scale bars denote 500 µm (top) and 600 µm (bottom). (**b**) Low magnification (top) and high magnification (bottom) images of Entorhinal Cortex depicting lack of retrograde labeling from aDG. Scale bars denote 500 µm (top) and 600 µm (bottom). (**c**) Low magnification (top) and high magnification (bottom) images of SUM depicting lack of retrograde labeling from aDG. Scale bars denote 500 µm (top) and 600 µm (bottom). (**d**) Low magnification (top) and high magnification (bottom) images of MS depicting lack of retrograde labeling from aDG. (**e**) Low magnification (top) and high magnification (bottom) images of PVN depicting lack of retrograde labeling from aDG. (**f)** Representative images of aDG hilus *Oxtr* mRNA expression in Oxtr +/+ and Oxtr f/f animals injected with hSyn-Cre virus. Scale bar denotes 50 µm. (**g**) Quantification of *Oxtr* mRNA in aDG expressed as a percentage of total DAPI+ cells in Oxtr +/+ and Oxtr f/f animals injected with hSyn-Cre virus.

Supplementary Figure 2

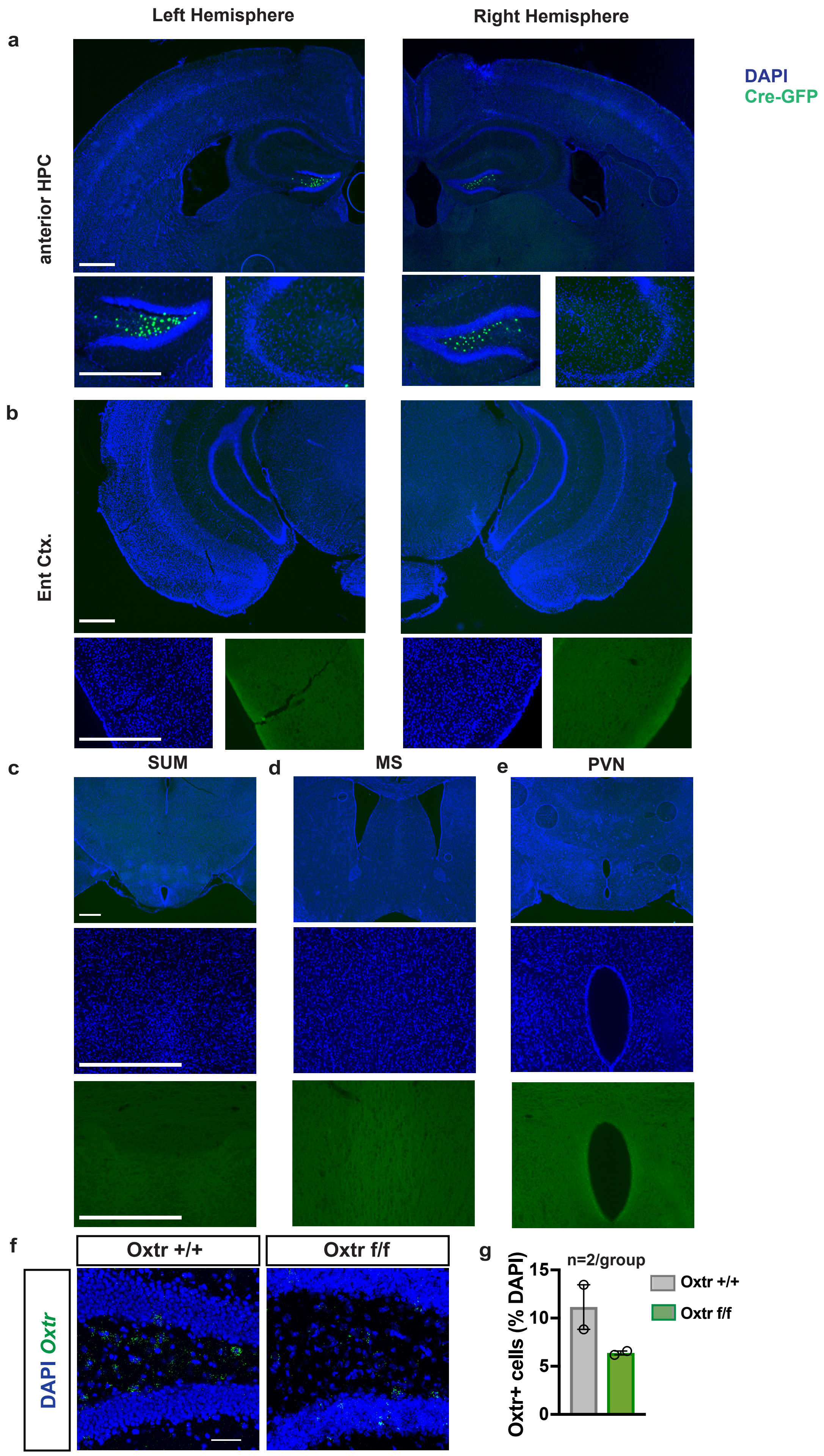

Supplementary Figure 3. Viral recombination of *Oxtrs* in anterior DG hilar neurons does not affect behavioral measures of innate anxiety.

(**a**) Schematic illustrating viral injection and behavioral testing timeline. (**b**) Schematic illustrating open field test (left) and quantification of total locomotion, percent distance in center, and time in center (right, GFP: n=7, Cre: n=4). (**c**) Schematic illustrating elevated plus maze (left) and quantification of time in open arm and time in closed arm (right, GFP: n=7, Cre: n=4). All data are displayed as mean ± SEM.

Supplementary Figure 3

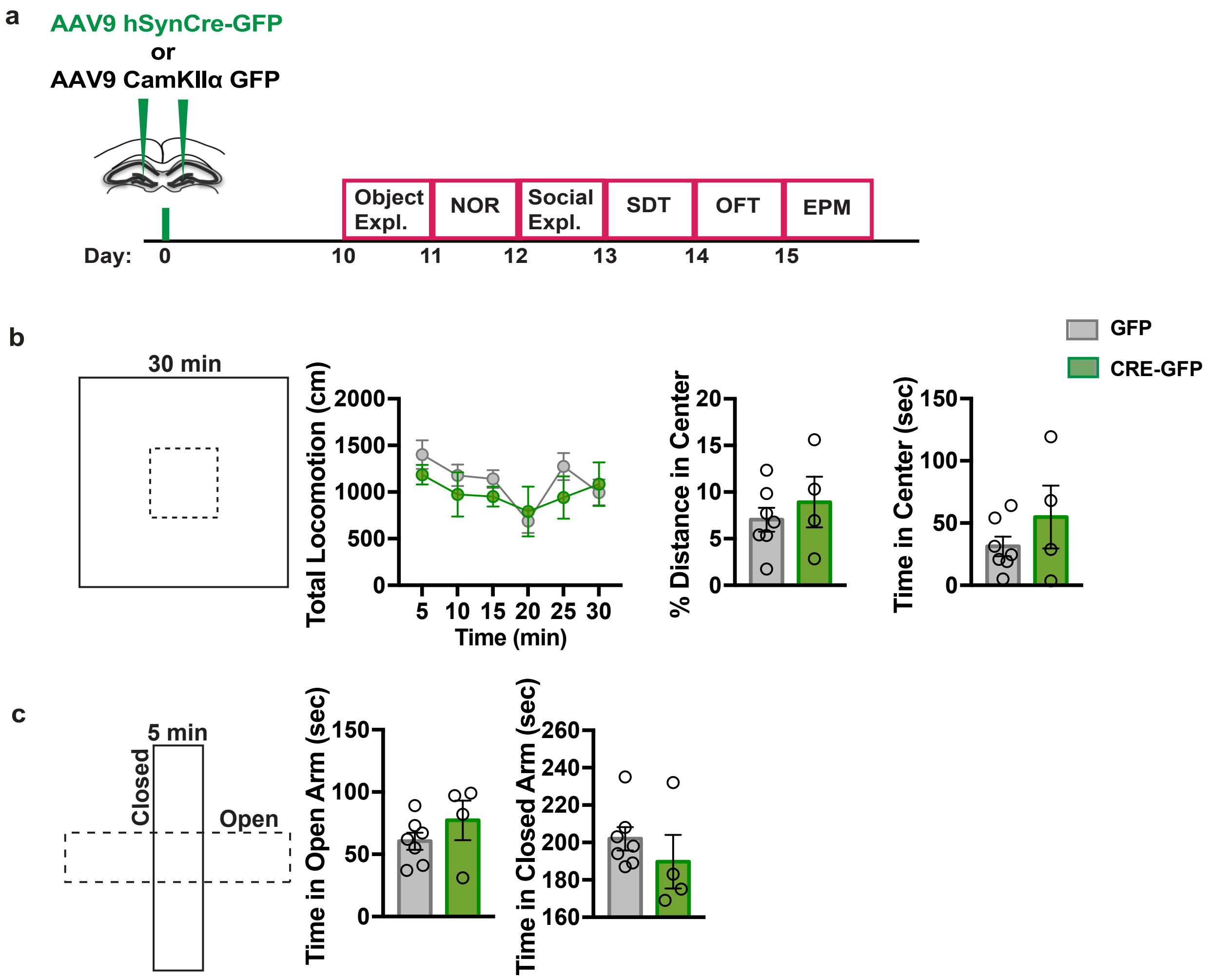

Supplementary Figure 4. AAV_9-CaMKIIα-Cre-GFP injected into $aCA2/CA3_{distal}$ is not retrogradely or anterogradely trafficked.

(**a**) Low magnification (top) and high magnification (bottom) images of anterior hippocampus at the site of injection in $aCA2/CA3_{distal.}$ Scale bars denote 500 μm (top) and 600 μm (bottom). (**b**) Low magnification (top) and high magnification (bottom) images of Entorhinal Cortex depicting lack of retrograde labeling from $aCA2/CA3_{distal}$. Scale bars denote 500 μm (top) and 600 μm (bottom). (**c**) Low magnification (left) and high magnification (right) images of PVN depicting lack of retrograde labeling from $aCA2/CA3_{distal}$. Scale bars denote 500 μm (left) and 600 μm (right). (**d)** Representative images of $aCA2/CA3_{distal}$ *Oxtr* mRNA expression in Oxtr +/+ and Oxtr f/f animals injected with CaMKIIα-Cre virus. Scale bar denotes 50 μm. (**e**) Quantification of *Oxtr* mRNA in $aCA2/CA3_{distal}$ expressed as a percentage of total DAPI+ cells in Oxtr +/+ and Oxtr f/f animals injected with CaMKIIα-Cre virus.

Supplementary Figure 4

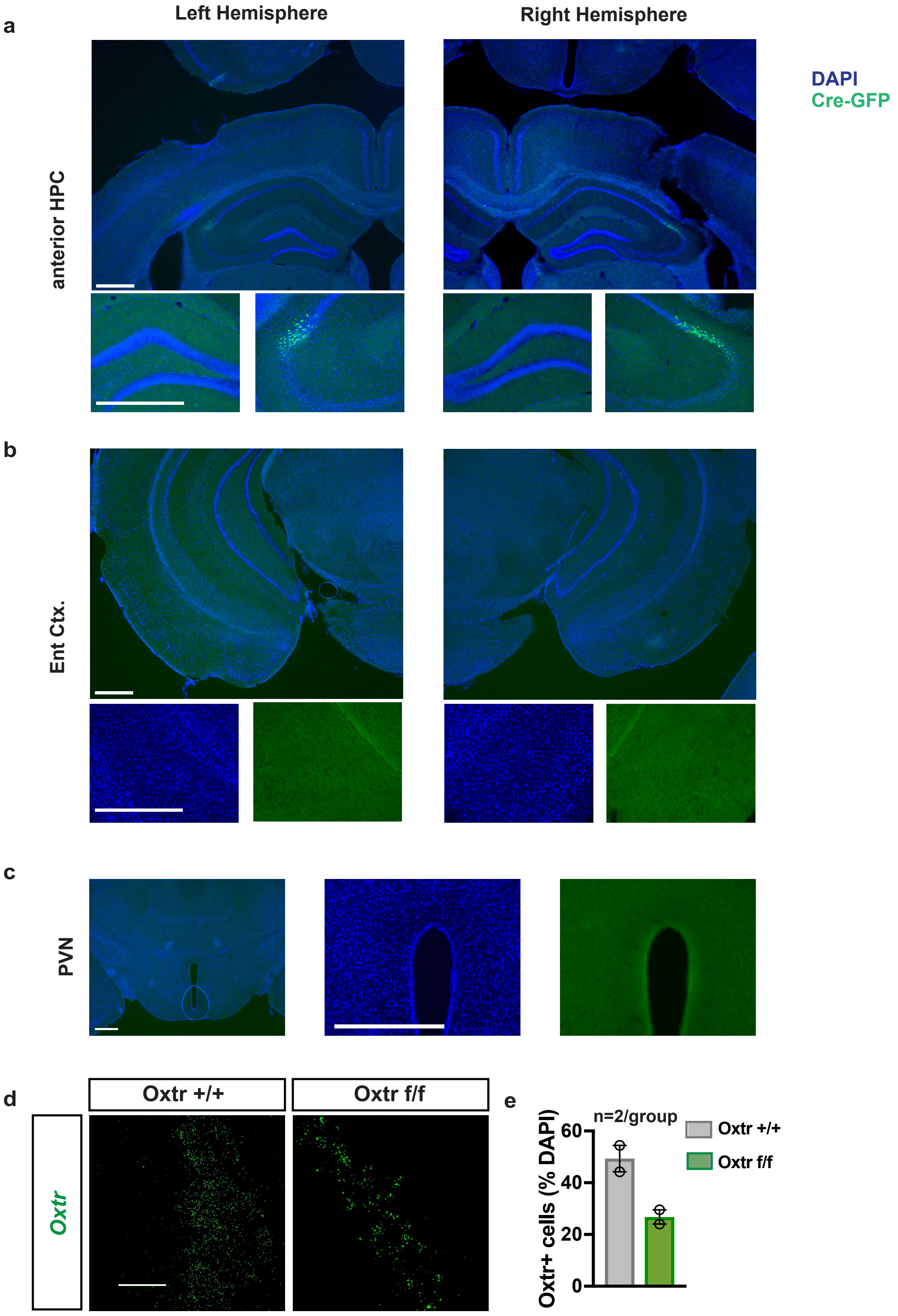

Supplementary Figure 5. Assessment of behavioral measures of innate anxiety following viral recombination of *Oxtrs* in aCA2/CA3$_{\mathbf{distal}}$.

(**a**) Schematic illustrating viral injection and behavioral testing timeline. (**b**) Representative image (left) and quantification (right) of overlap of *Cre* mRNA with *Gad1* mRNA. Scale bar denotes 50 µm. (**c**) Schematic illustrating open field test (left) and quantification of total locomotion, percent distance in center, and time in center (right, GFP: n=7, Cre: n=10). (**d**) Schematic illustrating elevated plus maze (left) and quantification of time in open arm, time in closed arm, and time in center (right, GFP: n=7, Cre: n=10). All data are displayed as mean ± SEM.

Supplementary Figure 5

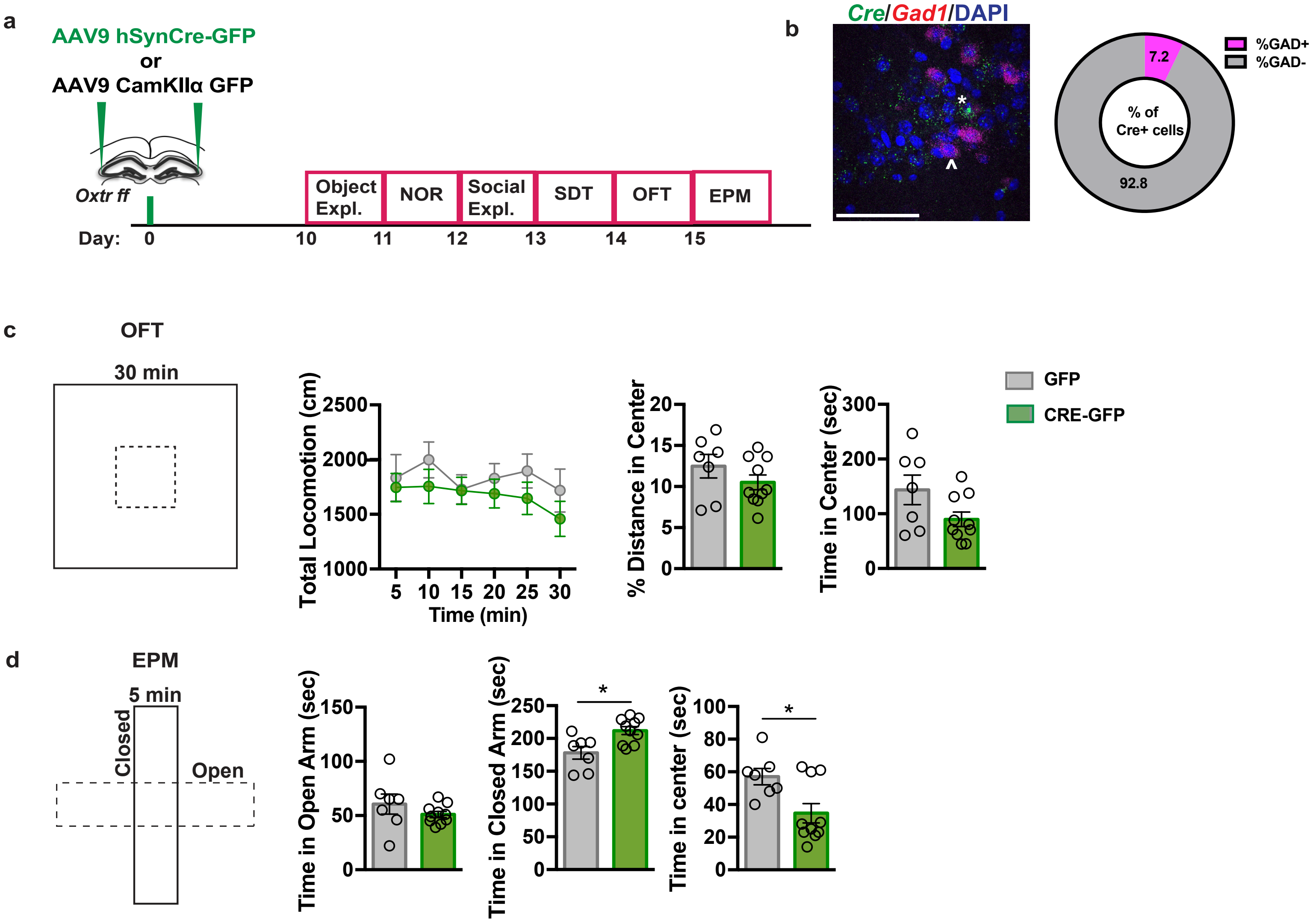

Supplementary Figure 6. Pharmacological blockade of Oxtrs in aCA2/CA3$_{distal}$ after acquisition is sufficient to impair retrieval.

(**a**) Schematic illustrating placement of guide cannulae and behavioral testing timeline. (**b**) Representative image of guide cannula placement above aCA2/CA3$_{distal}$. Arrowhead represents tract of cannula. Scale bar denotes 200 µm. (**c**) Quantification of social discrimination task (n=6). Quantifications are displayed as Habituation (trials 1-3), Test (trial 4), and discrimination ratio (trial 4). All data are displayed as mean ± SEM.

Supplementary Figure 6

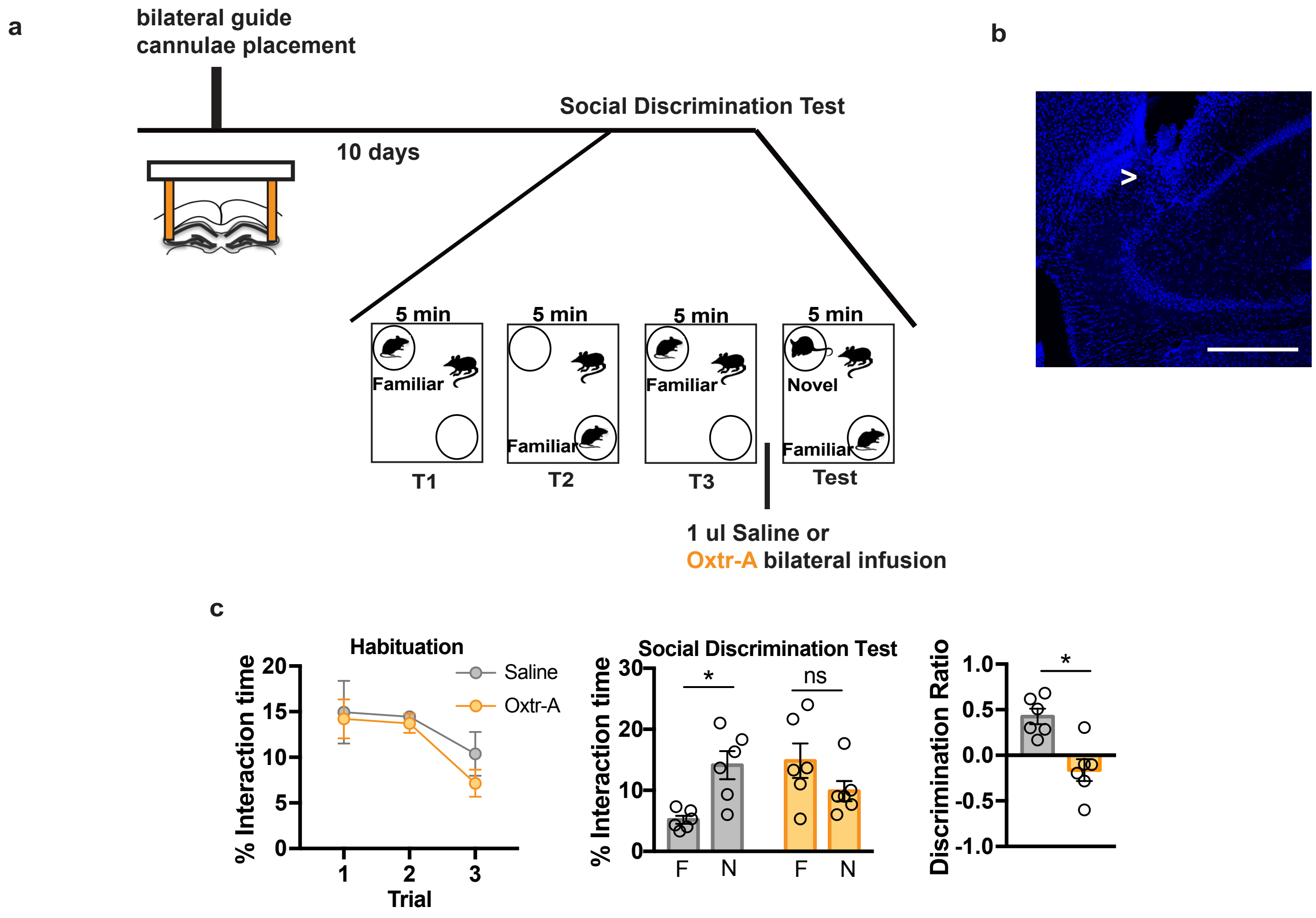

Supplementary Figure 7. Representative catFISH images.
Representative confocal images of $CA3_{proximal}$ (**a**) and $CA3_{distal}$ (**b**) exhibiting cytoplasmic, nuclear, or both (cytoplasmic and nuclear) localization of *cFos* transcripts. Scale bar denotes 50 μm.

Supplementary Figure 7

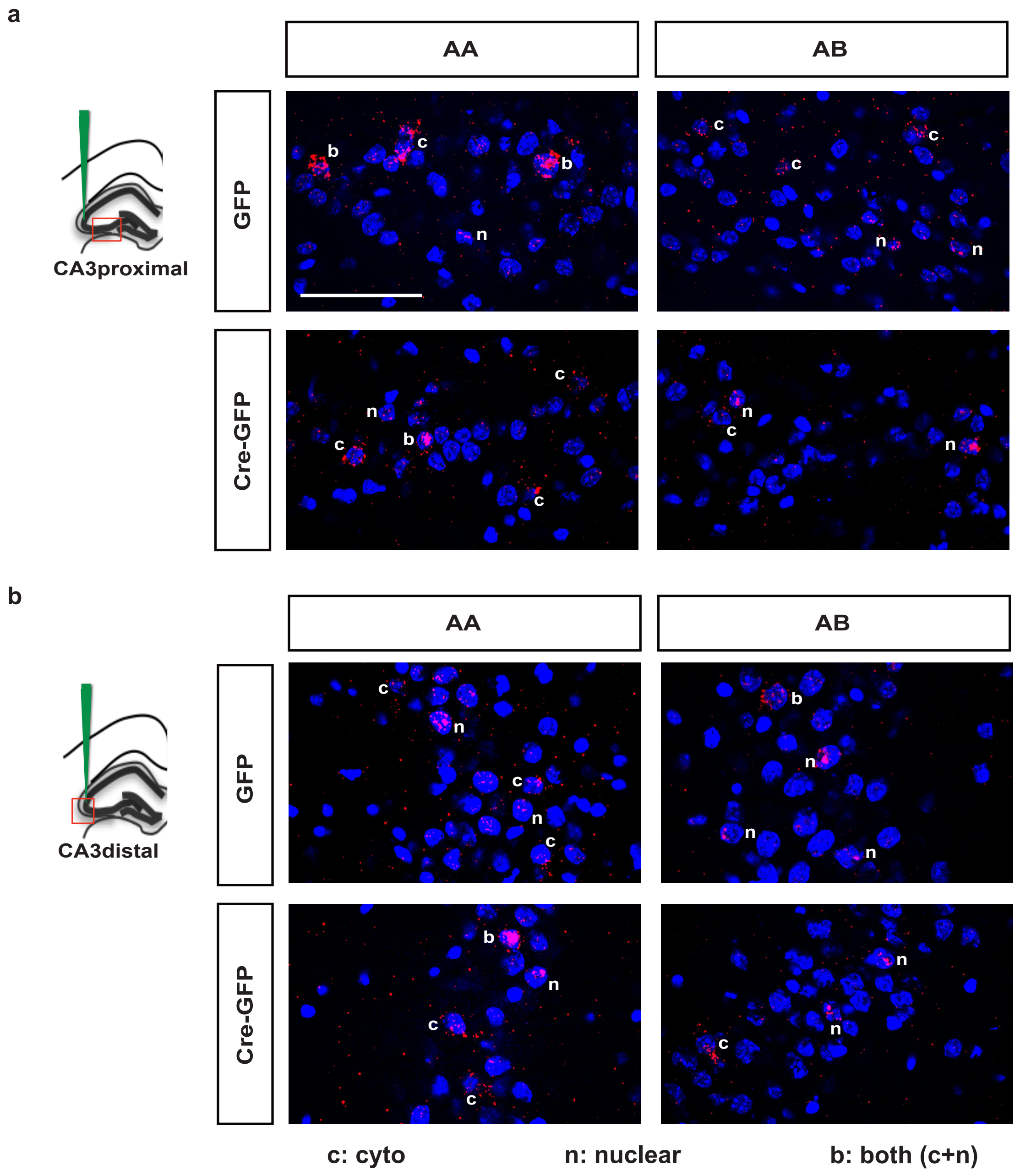

Supplementary Figure 8. Representative images of projection pattern from aCA2/$CA3_{distal}$ to aCA1, pCA1, and DLS.

(**a**) Representative low magnification image of injection of NpHR virus in aCA2/$CA3_{distal}$. Scale bar denotes 500 μm. (**b**) Representative low magnification (left) and high magnification (right) images of NpHR terminals in dorsolateral septum (DLS). Scale bars denote 500 μm (left) and 100 μm (right). (**c**) Representative low magnification images of NpHR terminals in posterior CA1. Scale bar denotes 500 μm.

Supplementary Figure 8

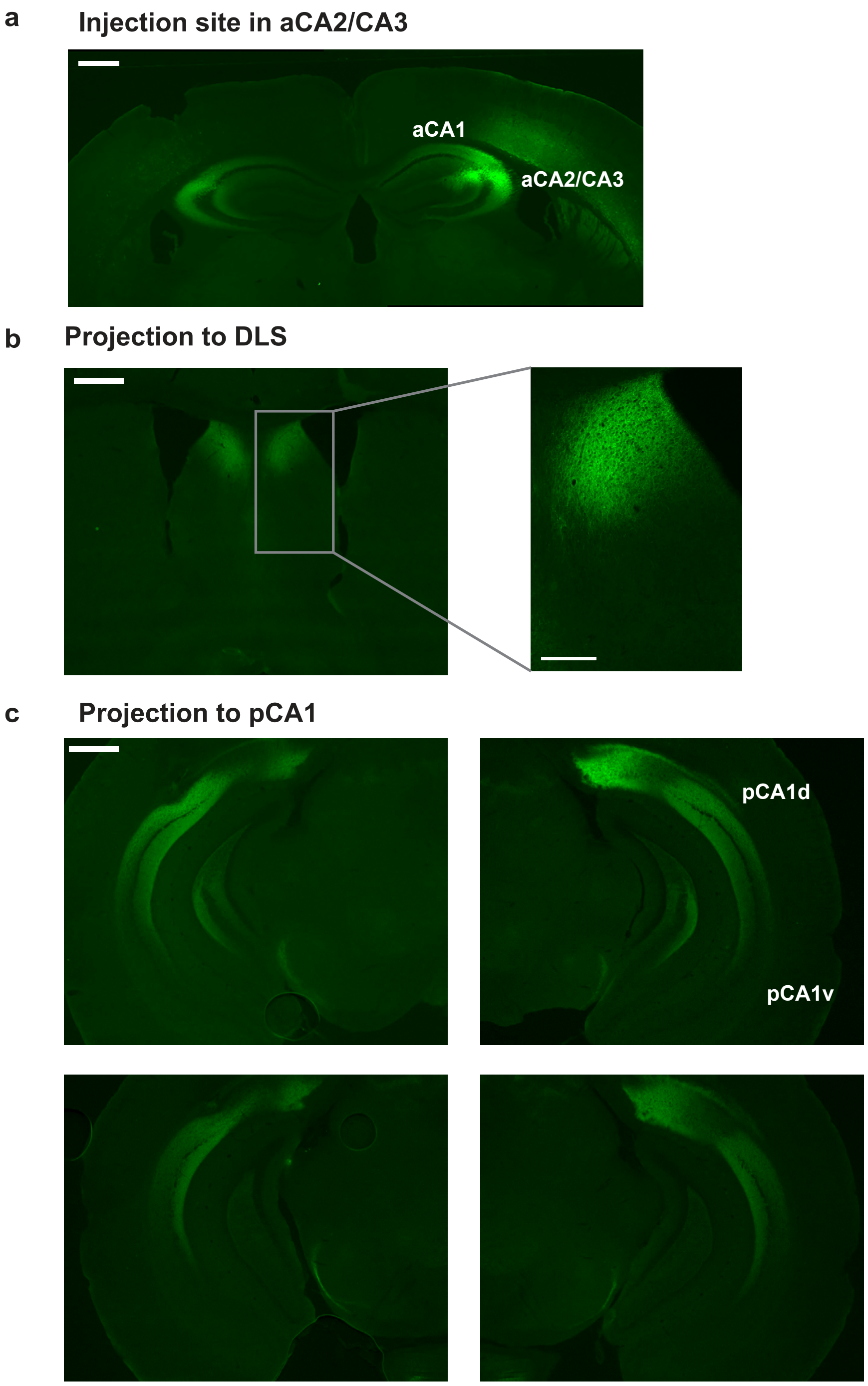

Supplementary Figure 9. Optogenetic attenuation of aCA2/CA3$_{distal}$ outputs to DLS modestly enhances discrimination of social stimuli.

(**a**) Schematic illustrating viral injection, optogentic implant and behavioral testing timeline. (**b**) Representative images of site of injection of eNpHR3.0 virus in aCA2/CA3$_{distal}$ cell bodies (top) and corresponding axon terminals in DLS (bottom). Scale bar denotes 200 µm. (**c**) Representative images and quantifications of cFos immunoreactivity in termination zone of DLS during optogenetic silencing (EYFP: n=11, NpHR: n=9). Scale bar denotes 200 µm. (**d**) Behavioral schematic (top) and quantification (bottom) of single object exploration (EYFP: n=17, NpHR: n=17). (**e**) Behavioral schematic (top) and quantification (bottom) of novel objection recognition (EYFP: n=14, NpHR: n=14). Quantifications are displayed as Habituation (trials 1-3), Test (trial 4), and discrimination ratio (trial 4). Laser was on during trial 4 only. (**f**) Behavioral schematic (top) and quantification (bottom) of social exploration test (EYFP: n=10, NpHR: n=10). (**g**) Behavioral schematic (top) and quantification (bottom) of social discrimination task (EYFP: n=17, NpHR: n=15). Quantifications are displayed as Habituation (trials 1-3), Test (trial 4), and discrimination ratio (trial 4). Laser was on during trial 4 only. All data are displayed as mean ± SEM.

Supplementary Figure 9

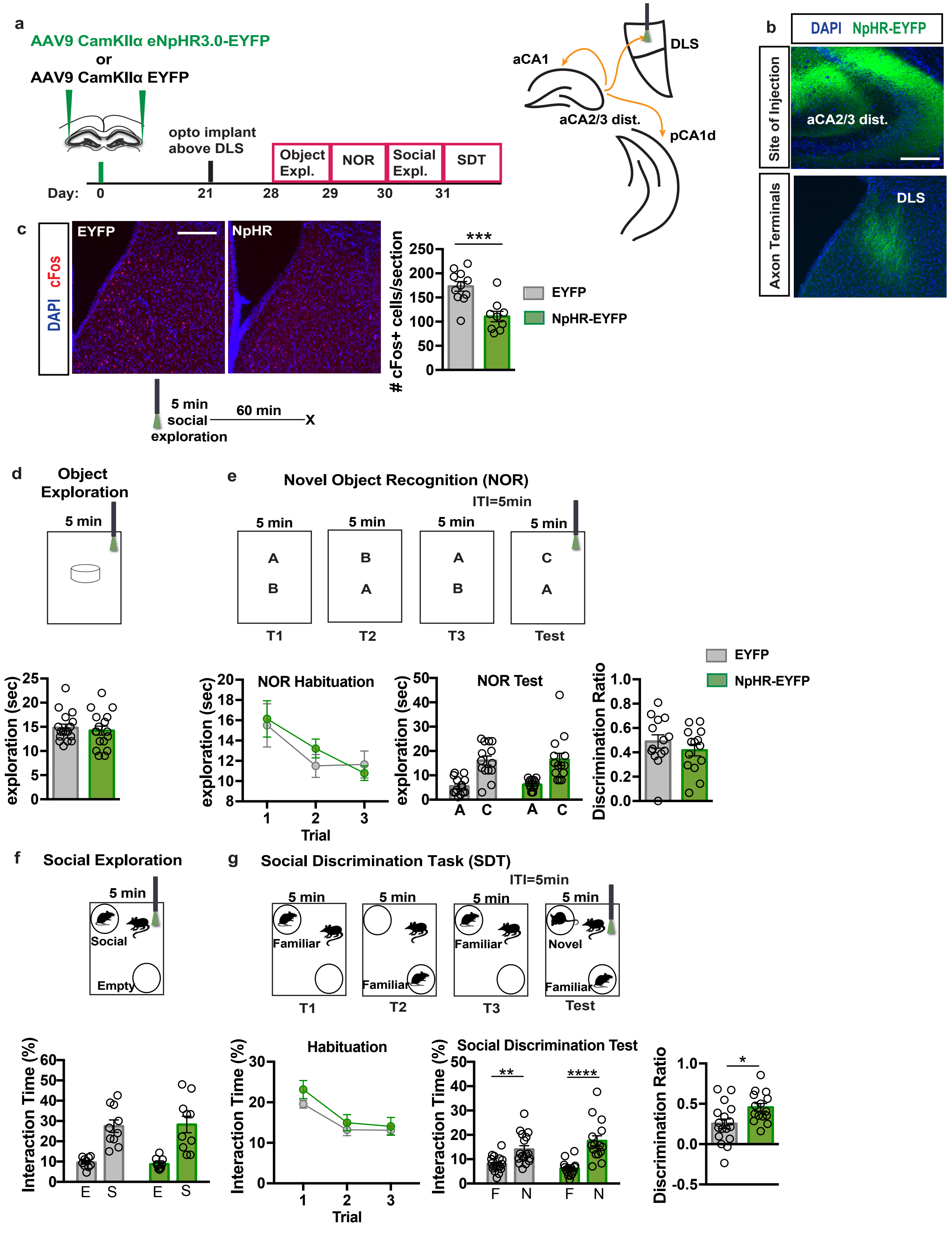

Supplementary Figure 10. Projections from aCA2/CA3$_{distal}$ colocalize with CA1 marker WFS1 in both anterior and posterior CA1.

(**a**) Low magnification (left) and high magnification (right) images of coronal sections depicting projections from aCA2/CA3$_{distal}$ to aCA1. Scale bars denote 500 µm (left) and 200 µm (right). (**b**) Low magnification (left) and high magnification (right) images of coronal sections depicting projections from aCA2/CA3$_{distal}$ to pCA1. Scale bars denote 500 µm (left) and 200 µm (right). (**c**) Representative images of horizontal sections from most superficial (1) to most deep (6), depicting projections from aCA2/CA3$_{distal}$ to aCA1 and pCA1d. Scale bar denotes 200 µm.

Supplementary Figure 10

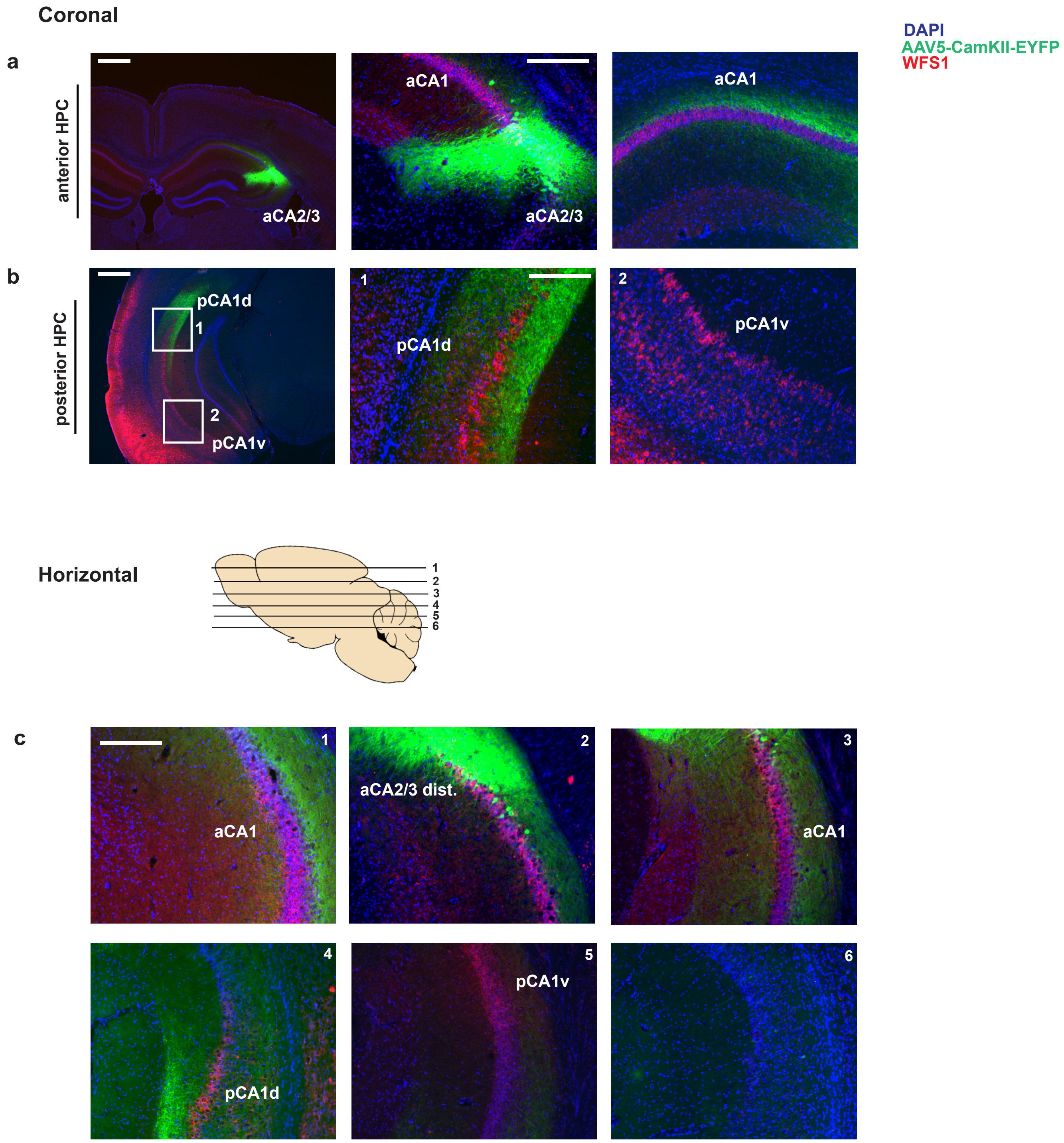

Supplementary Note 1

Open Field Test

Locomotor behavior was recorded for 30 min divided in six 5 min epochs in a Plexiglas open-field (OF) box of 41 x 41cm (Kinder Scientific) with 16 sets of double stacked pulse-modulated infrared photobeams (SmartFrame Open Field System; Kinder Scientific, Poway, CA) equally spaced on every wall (128 total) to record x-y ambulatory movements. MotorMonitor Software (Kinder Scientific, Poway,CA) defined grid lines that divided the open field into center (25% of total area) and periphery (75% of total area), with the periphery consisting of the 10 cm closest to the wall around the entire perimeter. Dependent measures were the overall motor activity quantified as the total locomotion (in centimeters), the distance traveled in the center divided by total distance traveled (percentage distance in center), and the time spent in the center (seconds). For *Oxtr f/f* DG cohort, two cohorts were used in order to increase Ns, and tests for innate anxiety (OFT and EPM) were performed only on the first cohort.

Elevated Plus Maze

Innate anxiety was recorded in the elevated plus maze (EPM) for 5 min. The maze consisted of a black Plexiglas apparatus placed 1m above the floor, with two open arms (67 cm x 7 cm) perpendicular to two enclosed arms (67 x 7 x 17 cm). The four arms were separated by a neutral transition central square (5 x 5 cm) in which mice were placed at the beginning of the experiment and their behavior was recorded for 5 min with a video camera system (ViewPoint, Lyon, France) located above the maze. Cumulative time spent in the open, closed, and center arms was scored manually by an investigator blind to the treatment conditions and data were expressed as the time spent in open arms (seconds), time spent in closed arms (seconds), and time spent in center (for $Oxtr^{f/f}$ aCA2/CA3$_{distal}$ cohort only). An arm visit was recorded when the mouse moved its forepaws into the arm. For $Oxtr^{f/f}$ DG cohort, two cohorts were used in order to increase Ns, and tests for innate anxiety (OFT and EPM) were performed only on the first cohort.

Pharmacological Blockade of Oxtrs

Cannula implantation for drug delivery to CA1 was carried out as described previously [47]. Cannula were purchased from Plastics One (Roanoke, VA) and consisted of bilateral guide cannula (center to center 5.0mm, custom depth 1.8 mm) and removable dummy cannula. Adult (8-10 week-old) C57Bl/6J male mice were maintained under standard housing conditions, and anaesthetized with ketamine / xylazine (10mg/mL and 1.6mg/mL, i.p.). Mice were placed in the stereotaxic apparatus and small hole was drilled at each bilateral injection location (AP = -1.9mm from bregma; ML = ± 2.5mm) and cannula slowly lowered to the appropriate depth. The cannula was secured to the skull with two cranial screws and dental cement. After allowing ten days for the mice to recover, the dummy cannula was removed, and the injection cannula was inserted through the guide cannula, from which it projected 0.5mm. The injection cannula was attached to PE50 tubing and a New Era Syringe pump. 1µl bilateral sterile saline or Oxtr antagonist, Oxtr-A, (L-368,899 Hydrochloride, Tocris #2641, dose 10 mM) was infused at a rate of 1µl/minute bilaterally. Infusion occurred directly after trial 3 and before trial 4 of the social discrimination task (inter-trial interval 10 min). This experiment followed a within-animal comparison design in which half the animals received saline treatment and half received Oxtr-A on the first day, and were subsequently counterbalanced for treatment on the following day.

Supplementary Table 1 Statistics details of data shown in main figures

Figure	Panel	Test Used		F (DFn, DFd)	P value	Post hoc
2	C	unpaired t-test		t=4.017 df=4.012	P=0.0158	
	D	unpaired t-test		t=0.1557 df=12.19	P=0.8788	
	E-Habituation	2way ANOVA	Interaction	F (2, 30) = 0.3453	P=0.7108	
			Time	F (2, 30) = 17.53	P<0.0001	
			Treatment	F (1, 15) = 0.003425	P=0.9541	
	E-Test	2way ANOVA	Interaction	F (1, 15) = 0.1703	P=0.6857	
			Treatment	F (1, 15) = 0.3658	P=0.5543	
			Stimulus	F (1, 15) = 18.72	P=0.0006	
	E-Discrimination Ratio	unpaired t-test		t=0.3992 df=8.889	P=0.6992	
	F	2way ANOVA	Interaction	F (1, 15) = 0.5433	P=0.4724	
			Treatment	F (1, 15) = 2.063	P=0.1714	
			Stimulus	F (1, 15) = 56.51	P<0.0001	
	G-Habituation	2way ANOVA	Interaction	F (2, 30) = 2.613	P=0.0899	
			Time	F (2, 30) = 4.568	P=0.0185	
			Treatment	F (1, 15) = 3.17	P=0.0953	Bonferroni
	G-Test	2way ANOVA	Interaction	F (1, 15) = 10.32	P=0.0058	
			Treatment	F (1, 15) = 0.8955	P=0.3590	
			Stimulus	F (1, 15) = 25.78	P=0.0001	
	G-Discrimination Ratio	unpaired t-test		t=3.989 df=8.408	P=0.0036	
3	C	unpaired t-test		t=0.4911 df=9.28	P=0.6347	
	D-Habituation	2way ANOVA	Interaction	F (2, 30) = 0.1332	P=0.8758	
			Time	F (2, 30) = 27.23	P<0.0001	
			Treatment	F (1, 15) = 0.5418	P=0.4730	
	D-Test	2way ANOVA	Interaction	F (1, 15) = 0.1795	P=0.6778	
			Treatment	F (1, 15) = 0.5954	P=0.4523	
			Stimulus	F (1, 15) = 125.2	P<0.0001	
	D-Discrimination Ratio	unpaired t-test		t=1.19 df=14.68	P=0.2529	
	E	2way ANOVA	Interaction	F (1, 14) = 0.1853	P=0.6734	
			Treatment	F (1, 14) = 1.256	P=0.2813	
			Stimulus	F (1, 14) = 64.07	P<0.0001	
	F-Habituation	2way ANOVA	Interaction	F (2, 30) = 6.282	P=0.0053	Bonferroni
			Time	F (2, 30) = 0.6646	P=0.5219	
			Treatment	F (1, 15) = 0.2574	P=0.6193	
	F-Test	2way ANOVA	Interaction	F (1, 15) = 5.597	P=0.0319	Bonferroni
			Treatment	F (1, 15) = 0.001845	P=0.9663	
			Stimulus	F (1, 15) = 19.98	P=0.0004	
	F-Discrimination Ratio	unpaired t-test		t=3.499 df=13.32	P=0.0038	

4	D	2way ANOVA	Interaction	F (1, 15) = 5.917	P=0.0280	Bonferroni
			Treatment	F (1, 15) = 0.1703	P=0.6856	
			Exposure	F (1, 15) = 3.335	P=0.0878	
	E	2way ANOVA	Interaction	F (1, 15) = 0.8261	P=0.3778	
			Treatment	F (1, 15) = 1.643	P=0.2194	
			Exposure	F (1, 15) = 1.977	P=0.1801	
	F	2way ANOVA	Interaction	F (1, 15) = 0.1074	P=0.7477	
			Treatment	F (1, 15) = 0.3531	P=0.5612	
			Exposure	F (1, 15) = 0.5867	P=0.4556	
	G	2way ANOVA	Interaction	F (1, 15) = 1.607	P=0.2242	
			Treatment	F (1, 15) = 0.5028	P=0.4891	
			Exposure	F (1, 15) = -5.271e-014	P>0.9999	
5	C	unpaired t-test		t=0.1049 df=13.79	P=0.918	
	D-Habituation	2way ANOVA	Interaction	F (2, 28) = 0.4736	P=0.6276	
			Time	F (2, 28) = 7.169	P=0.0031	
			Treatment	F (1, 14) = 2.006	P=0.1786	
	D-Test	2way ANOVA	Interaction	F (1, 14) = 15.88	P=.0014	Bonferroni
			Treatment	F (1, 14) = 3.783	P=.0721	
			Stimulus	F (1, 14) = 28.67	P=.0001	
	D-Discrimination Ratio	unpaired t-test		t=3.535 df=12.2	P=0.0029	
	E	2way ANOVA	Interaction	F (1, 15) = 0.008932	P=0.9260	
			Treatment	F (1, 15) = 2.244	P=0.1548	
			Stimulus	F (1, 15) = 196.3	P<0.0001	
	F-Habituation	2way ANOVA	Interaction	F (2, 20) = 1.463	P=0.2553	
			Time	F (2, 20) = 14.24	P=0.0001	
			Treatment	F (1, 10) = 0.07274	P=0.7929	
	F-Test	2way ANOVA	Interaction	F (1, 10) = 0.3889	P=0.5469	
			Treatment	F (1, 10) = 0.4151	P=0.5339	
			Stimulus	F (1, 10) = 35.89	P=0.0001	
	F-Discrimination Ratio	unpaired t-test		t=0.5153 df=9.619	P=0.618	
6	C	unpaired t-test		t=2.907 df=7.124	P=0.0223	
	D	unpaired t-test		t=1.424 df=11.18	P=0.1818	
	E-Habituation	2way ANOVA	Interaction	F (2, 22) = 0.6386	P=0.5376	
			Time	F (2, 22) = 9.01	P=0.0014	
			Treatment	F (1, 11) = 0.1533	P=0.7029	
	E-Test	2way ANOVA	Interaction	F (1, 11) = 0.1275	P=0.7278	
			Treatment	F (1, 11) = 0.1941	P=0.6681	
			Stimulus	F (1, 11) = 37.73	P<0.0001	
	E-Discrimination Ratio	unpaired t-test		t=1.873 df=10.04	P=0.0904	
	F	2way ANOVA	Interaction	F (1, 12) = 2.302	P=0.1551	

			Treatment	F (1, 12) = 0.8622	P=0.3714	
			Stimulus	F (1, 12) = 147.3	P<0.0001	
	G-Habituation	2way ANOVA	Interaction	F (2, 24) = 0.5903	P=0.5620	
			Time	F (2, 24) = 19	P<0.0001	
			Treatment	F (1, 12) = 0.6627	P=0.4315	
	G-Test	2way ANOVA	Interaction	F (1, 12) = 13.4	P=0.0033	Bonferroni
			Treatment	F (1, 12) = 0.82	P=0.3830	
			Stimulus	F (1, 12) = 64.71	P<0.0001	
	G-Discrimination Ratio	unpaired t-test		t=4.664 df=11.29	P=0.0006	
S3	B-Total Locomotion	2way ANOVA	Interaction	F (5, 45) = 0.9364	P=0.4667	
			Time	F (5, 45) = 3.739	P=0.0065	
			Treatment	F (1, 9) = 0.6418	P=0.4437	
	B-% Distance Center	unpaired t-test		t=0.633 df=4.409	P=0.5581	
	B-Time in Center	unpaired t-test		t=0.8953 df=3.592	P=0.4266	
	C-Time Open Arm	unpaired t-test		t=0.9644 df=4.152	P=0.3876	
	C-Time Closed Arm	unpaired t-test		t=0.7832 df=4.138	P=0.4759	
S5	C-Total Locomotion	2way ANOVA	Interaction	F (5, 75) = 0.5447	P=0.7418	
			Time	F (5, 75) = 1.894	P=0.1055	
			Treatment	F (1, 15) = 0.838	P=0.3744	
	C-% Distance Center	unpaired t-test		t=1.165 df=10.58	P=0.2698	
	C-Time in Center	unpaired t-test		t=1.788 df=8.839	P=0.1081	
	D-Time Open Arm	unpaired t-test		t=1.008 df=7.101	P=0.3465	
	D-Time Closed Arm	unpaired t-test		t=2.957 df=10.54	P=0.0136	
	D-Time in Center	unpaired t-test		t=2.891 df=14.99	P=0.0112	
S6	C-Habituation	2way ANOVA	Interaction	F (2, 20) = 0.3557	P=0.7051	
			Time	F (2, 20) = 7.068	P=0.0048	
			Treatment	F (1, 10) = 0.5164	P=0.4888	
	C-Test	2way ANOVA	Treatment	F (1, 5) = 2.373	P=0.1841	
			Stimulus	F (1, 5) = 1.509	P=0.2739	
			Interaction: Group x	F (1, 5) = 9.802	P=0.0259	paired t-test
	C-Discrimination Ratio	paired t-test		t=3.948 df=5	P=0.0109	
S9	C	unpaired t-test		t=4.197 df=17.44	P=0.0006	
	D	unpaired t-test		t=0.5006 df=30.66	P=0.6202	
	E-Habituation	2way ANOVA	Interaction	F (2, 52) = 0.496	P=0.6118	
			Time	F (2, 52) = 6.845	P=0.0023	
			Treatment	F (1, 26) = 0.1365	P=0.7148	
	E-Test	2way ANOVA	Interaction	F (1, 26) = 0.007116	P=0.9334	
			Treatment	F (1, 26) = 0.07521	P=0.7861	
			Stimulus	F (1, 26) = 66.96	P<0.0001	
	E-Discrimination Ratio	unpaired t-test		t=0.9804 df=25.56	P=0.3361	

F	2way ANOVA	Interaction	F (1, 18) = 0.108	P=0.7462	
		Treatment	F (1, 18) = 2.407e-013	P>0.9999	
		Stimulus	F (1, 18) = 54.41	P<0.0001	
G-Habituation	2way ANOVA	Interaction	F (2, 60) = 1.087	P=0.3437	
		Time	F (2, 60) = 44.98	P<0.0001	
		Treatment	F (1, 30) = 0.9765	P=0.3310	
G-Test	2way ANOVA	Interaction	F (1, 30) = 4.861	P=0.0353	Bonferroni
		Treatment	F (1, 30) = 0.2753	P=0.6037	
		Stimulus	F (1, 30) = 52.65	P<0.0001	
G-Discrimination Ratio	unpaired t-test		t=2.674 df=29.36	P=0.0121	
